# Supplementary material for: Secondary Metabolites Isolated from Artemisia afra and Artemisia annua and Their Anti-Malarial, Anti-Inflammatory and Immunomodulating Properties—Pharmacokinetics and Pharmacodynamics: A Review
Source: Metabolites. 2023 Apr 29;13(5):613. doi: 10.3390/metabo13050613 (PMC10222324; doi:10.3390/metabo13050613)
Supplement: Supplementary file 1 [file metabolites-13-00613-s001.zip › metabolites-2316633-Supplementary material.pdf]

Supplementary material.

Table S1: Monoterpenes isolated from *A. annua* and *A. afra.*

N	Compound	*A. annua*	*A. afra*	Reference
1	α- myrcene hydroperoxide	P	A	[135]
2	(-)-Myrtenyl acetate	P	A	[136]
3	β-myrcene hydroperoxide	P	A	[135]
4	β-Sabinene hydrate	P	A	[137]
5	(*E*)- 3,7-Dimethyl-1,3,6-octatriene	P	A	[137]
6	(*E*)-2-Butenoic acid, 2-methyl-, 2,2-dimethyl-1-(2-methyl-1-propenyl)- 3-buteyl ester	P	A	[137]
7	(*E*)-7-hydroperoxy-2,7-dimethylocta-2,5-dien-4-one	P	A	[23]
8	(*E*)-7-hydroxy-2,7-dimethylocta- 2,5-dien-4-one	P	A	[23]
9	(*E*)-Isobutyric acid	P	A	[138]
10	1,10-Oxy-α-myrcene hydroxide	P	A	[135,139]
11	1,10-Oxy-β-myrcene hydroxide	P	A	[135]
12	1,4-cineole	P	A	[6]
13	1,8-cineole	p	p	[66,140]
14	2-Butenoic acid, 3-methyl- (1*S*,2*R*,4*S*)-1,7,7-trimethylbicyclo[2.2.1]hept-2-yl ester	P	A	[141]
15	2-Cyclohexen-1-one, 2-methyl-5-(1-methylcyclopropyl)	P	A	[137]
16	2-methyl butyl isovalerate	A	P	[139]
17	2-methyl butyl isovalerate	A	P	[139]
18	2-methyl-6- methylene- 1,7-Octadien-3-one	P	A	[141]
19	2-α-Hydroxy-1,8-cineole	P	A	[142]
20	2,3-Dihydro-1,8-cineole	P	P	[137,139,140]
21	2,6-Dimethyl-1,3,5,7-octatetraene	P	A	[138]
22	2,6-Dimethyl-1,5,7-octatrien-3-ol	P	A	[136]
23	2,6-Dimethyl-3,5,7-octatrien-2-ol	P	A	[136]
24	2,6-Dimethyl-3,5,7-octatrien-2-ol	P	A	[137,143]
25	2,6-Octadien-1-ol, 2,6-dimethyl-8-[(tetrahydro-2*H*-pyran-2-yl) oxy]	P	A	[136]
26	2,6,6- trimethyl-4-methylene Bicyclo[3.1.1]heptan-3-one	P	A	[40]
27	3-Cyclohexene-1-methanol 2-hydroxy-α,α,4-trimethyl-, 1-acetate	P	A	[144]
28	3-Pinanol	P	A	[145]
29	3-thujanone	P	P	[145,146]
30	3-Thujen-10-al	P	A	[147]
31	3-Thujen-2-ol	P	A	[6]
32	3,7-Dimethyl-1,5,7-octatrien-3-ol	P	A	[148]
33	3,7-Octadien-2-ol, 2-methyl-6-methylene	P	A	[141]
34	3,7-Octadien-2-ol, 2,6-dimethyl	P	A	[136]
35	4-Hydroxy-2-isopropenyl-5- methylene-hexan-1-ol	P	A	[135]
36	4-Terpineol	P	P	[137,149]
37	4-Terpinyl acetate	P	A	[150]
38	4α-hydroxy achipendol	A	P	[139]
39	4β-hydroxy achipendol	A	P	[139]

40	6- hydroxy-γ-humulene	P	A	[23]
41	6,7-epoxy-6,7-dihydro-β-farnesene	P	A	[23]
42	7-methyl-3- methylene-1,6-Octadien-4-one	P	A	[137,142]
43	Allo-ocimene	P	P	[151]
44	Arteannuin P	P	A	[23]
45	Arteannuin Q	P	A	[23]
46	Artemisia alcohol	P	P	[139,152]
47	Artemisia ketone	P	P	[139,153]
48	Artemisiatriene	P	A	[136]
49	Artemisyl acetate	P	P	[139,147]
50	Ascaridole	P	P	[139,146]
51	Azulene	A	P	[66]
52	Borneol	p	p	[66,137]
53	Borneol isobutyrate	P	A	[137]
54	Bornyl acetate	P	A	[137,147]
55	Bornyl valerate	P	A	[141]
56	Camphene	A	P	[139]
57	Camphor	P	p	[139,144]
58	Carvacrol	P	A	[137]
59	Carvone	p	p	[144]
60	Caryophylla-2(12),6(13)- dien-5-one	A	P	[139]
61	Chrysanthenone	P	P	[137,139,152]
62	Cis-1,2-epoxy- terpinen-4-ol	A	P	[139]
63	Cis-2,7-dimethyl-4- octene-2,4-diol	A	P	[154]
64	Cis-carveol	P	P	[145,149,153]
65	Cis-carvyl acetate	P	A	[140]
66	Cis-chrysanthenol	P	P	[139,140]
67	Cis-chrysanthenyl acetate	P	P	[139,140]
68	Cis-*e*poxyocimene	P	A	[138]
69	Cis-p-menth-2-en-1-ol	P	P	[137,144]
70	Cis-*p*-Menth-2-en-1-ol	P	A	[140,147]
71	Cis-p-mentha-1-(7), 8-dien-2-ol	P	P	[137,139]
72	Cis-pinocarveol	P	A	[40]
73	Cis-sabinene hydrate	P	P	[139], [140]
74	Citronellal	P	A	[146]
75	Citronellol	P	A	[6]
76	Cuminal	P	A	[144,147]
77	Cuminic alcohol	P	P	[139,155]
78	Cyclopentanecarboxylic acid, 3-methylene-, 1,7,7-trimethylbicyclo- [2.2.1] hept-2-yl ester	P	A	[141]
79	Dehydro carvyl acetate	P	P	[139,147]
80	Dehydrosabinaketone	A	P	[139]
81	Endo-dehydronorborneo	P	A	[136]
82	Furanoid trans-p-menth-2,8- dien-1-ol	A	P	[139]
83	Geraniol	P	P	[156]
84	Geranyl acetate	P	A	[136,40,156]
85	Ipsdienol	P	A	[147]
86	Iso-menthone	P	A	[141]
87	Isoamyl isovalerate	A	P	[139]
88	Isobornyl acetate	P	A	[140]
89	Isopiperitone	A	P	[153]

90	Lavandulane	P	A	[157]
91	Lavandulol	P	P	[135,139]
92	Lavandulyl acetate	P	A	[140]
93	Lavanduyl acetate	P	P	[135,139]
94	Lemonene	P	P	[40,139]
95	Limonene-1,2-epoxide	P	A	[147,155]
96	Linalol	A	P	[139]
97	Linalool acetate	A	P	[145]
98	Linalyl acetate	P	A	[137]
99	Menthol	P	A	[144,155]
100	Myrcene	P	P	[138,144]
101	Myrcenol	P	A	[40]
102	Myrtenal	P	P	[144,152]
103	Myrtenol	P	P	[140,144]
104	Nerol	P	A	[136]
105	Neryl acetate	P	A	[147]
106	*p*-Cymen-8-ol	P	P	[137]
107	*p*-Cymene	P	P	[139,148]
108	*p*-Menth-1-en-5-ol	P	A	[155]
109	*p*-Menth-2,8-dien-1-ol	P	A	[137]
110	*p*-Menth-3-ene	P	A	[138]
111	P-mentha-1,4-dien-7-ol	A	P	[139]
112	*p*-Mentha-1,4(8)-dien-3-ol	P	A	[137]
113	P-mentha-1,8-dien-10-ol	P	A	[137]
114	P-mentha-1,8-dien-10-ol	A	P	[139]
115	*p*-Mentha-1(7),5-dien-2-ol	P	A	[137]
116	*p*-Mentha-1(7),8-dien-2-ol	P	P	[151]
117	*p*-Mentha-2,4-diene	P	A	[144]
118	P-menthatriene	A	P	[156]
119	Perillaldehyde	P	A	[147]
120	Perillene	P	A	[136]
121	Phellandral	P	A	[136]
122	Pinocarvone	P	P	[139,140]
123	Pinocarvyl acetate	P	A	[40]
124	Piperitol	P	P	[6,144]
125	Piperitone	P	P	[6,139]
126	Sabina ketone	P	P	[123,126]
127	Sabinene	P	P	[139,151]
128	Sabinol	P	A	[137]
129	Sabinyl acetate	P	P	[139,140,151]
130	Santolina alcohol	P	A	[140,151]
131	Santolinatriene	P	A	[138,151]
132	Terpinen-4-ol	p	p	[139]
133	Terpinolene	P	P	[137,139]
134	Thymol	P	A	[137]
135	Trans- pinocarveol	P	P	[137,139]
136	trans-5-Hydroxy-2-isopropenyl-5-methylhex-3-en-1-ol	P	A	[157]
137	trans-Carvyl acetate	P	A	[140]
138	trans-Chrysanthenol	P	A	[140]
139	Trans-linalool oxide	A	P	[66]
140	Trans-p-menth-1(7), 8-dien-2-ol	A	P	[139]

141	Trans-p-meth-2-en-1-ol	P	P	[144]
142	Trans-piperitone oxide	P	P	[6,139]
143	Trans-sabinene hydrate	P	P	[139,140]
144	Trans-sabinol	a	p	[139]
145	trans-Sabinyl acetate	P	A	[137]
146	Trans-α-ocimene	P	P	[145,151]
147	Verbenone	P	A	[136]
148	Verbenyl acetate	P	A	[136]
149	Yomogi alcohol	P	A	[137]
150	(Z)-3,7-Dimethyl-1,3,6-octatriene	P	A	[137,139,140]
151	α-Fenchene	A	P	[144]
152	α-Myrcene hydroperoxide/β-myrcene hydroperoxide	P	A	[135]
153	α-Phellandrene	P	A	[137]
154	α-pinene	P	P	[137,145]
155	α-Terpinene	P	P	[139,158]
156	α-Terpineol	P	P	[146,148]
157	α-Thujene	P	P	[139,159]
158	α-Thujone	p	p	[138,139]
159	β-Phellandrene	p	p	[145,147]
160	β-Pinene	P	P	[40,139,140]
161	β-Pinene oxide	P	A	[140]
162	β-Thujene	p	p	[146,159]
163	β-Thujone	p	p	[138]
164	γ-Terpinene	P	P	[137,139]
165	δ-Terpineol	P	P	[139,140]

P = Present, A = Absent

Table S2: Sesquiterpenes from *A. annua* and *A. afra*

N	Compound	*A. annua*	*A. afra*	Reference
166	(-)-Amorpha-4,11-diene	P	A	[160]
167	(-)-Spathulenol	P	P	[139,140,147]
168	α-Bisabolol	P	P	[137,139]
169	δ-Cadinene	P	A	[6,141]
170	α-Cadinene	P	P	[152]
171	α-Cadinol	P	A	[137]
172	γ-Caryophyllene	p	P	[141]
173	β-Elemene	P	A	[6,19]
174	γ-Elemene	P	A	[141]
175	δ-Elemene	P	A	[141]
176	α-Epoxy-dihydroartemisinic	P	A	[161]
177	β-Hydroxy-4(15),7-eudesmadiene	P	A	[162]
178	δ-Muurolene	P	A	[137]
179	β-Selinene	p	P	[6,139]
180	(+)-Germacrene A	P	A	[163]
181	(1*R*,3*Z*,9*S*)-Bicyclo[7.2.0]undec-3-ene, 4,11,11-trimethyl-8-methylene-	P	A	[138]
182	(E)-nerolidol	A	P	[139]
183	(*E*)-Nerolidyl acetate	P	A	[164]
184	(*Z*)-1,3(15),6,10-Farnesatetraene	P	A	[122,123]

185	(Z)-7-acetoxy-methyl-11-methyl-3-methylenedodeca-1,6,10-triene	P	A	[165]
186	(Z)-jasmone	A	P	[139]
187	1-epi-cubenol	A	P	[139]
188	1-octen-3-ol	A	P	[132]
189	1-Oxo-2β-[3-butanone]-3α- methyl-6β-[2-propanoic acid]- cyclohexane	P	A	[162]
190	1-Oxo-2β-[3-butanone]-3α- methyl-6β-[2-propanol formyl ester]-cyclohexane	P	A	[162]
191	1α-Aldehyde-2β-[3-butanone]-3α- methyl-6β-[2-propanoic acid]- cyclohexane	P	A	[162]
192	1α-Aldehyde-2β-[3-butanone]-3α- methyl-6β-[2-propenoic acid]- cyclohexane	P	A	[162]
193	1β-Hydroxy-4(15),5-eudesmadiene	P	A	[162]
194	1β-Hydroxy-4(15),5(*E*),10(14)-germacratriene	P	A	[162]
195	1β,6α-Dihydroxy-4(15)-eudesmane	P	A	[162]
196	10-*epi*-γ-Eudesmol	P	A	[137]
197	11-hydroxy-arteannuin I	P	A	[23]
198	11*R*-(-)-Dihydroartemisinic acid	P	A	[137]
199	12α,4α- dihydroxybishopsolicepolide	A	P	[166]
200	14-Hydroxy-δ-cadinene	P	A	[137]
201	14-Hydroxy-α-humulene	P	A	[137]
202	15-*nor*-10-Hydroxy-oplopan-4-oic acid	P	A	[162]
203	2-Naphthalenol, decahydro-1-methyl-6-methylene-4-(1-methylethenyl)	P	A	[152]
204	2,3-Epoxy-7,10-bisaboladiene	P	A	[146]
205	2,6,10-Farnesatrien-1-ol acetate	P	A	[164]
206	2,7,10-Bisabolatriene	P	A	[167]
207	3-(2-(2,5-dihydrofuran-3-yl) ethyl)-2,2-dimethyl-4- methylenecyclohexan-1-one	P	A	[23]
208	3-Cedren-12-ol	P	A	[137]
209	3-Isobutylcadin-4-en-11-ol	P	A	[152]
210	3α-Hydroxy-4α,5α-epoxy-7-oxo- (8[7→6]-abeo-amorphane	P	A	[162]
211	3α,15-Dihydroxy cedrane	P	A	[162]
212	3α -hydroxydesoxy artemisinin	P	A	[6]
213	3α-15-dihydroxycedrane	P	A	[162]
214	3α, 7α -Dihydroxy-cadin-4-ene	P	A	[168]
215	4-Amorphen,3,11-diol	P	A	[152]
216	4-Amorphene-3,7-diol (3α,7α)			[168]
217	4-hydroxy-4 methylcyclohex- 2-enone	A	P	[139]
218	4-Muurolen-10-ol	P	A	[6]
219	4,7(11)-Amorphadien-12-al	P	A	[152]
220	4α,5α-Epoxy-6α-hydroxy amorphan-12-oic acid	P	A	[161]
221	4(15),11-Amorphadien-9-one	P	A	[152]
222	4(15),11-Eudesmadien-5α-ol	P	A	[162]
223	4(15),5,11-Cadinatriene	P	A	[152]
224	4α, 5α-epoxy-6α- hydroxyartemisinic acid methyl ester	P	A	[23]
225	5α -Hydroxy-eudesma-4(15), ll-diene	P	A	[168]
226	5α-Hydroperoxy-eudesma-4(15),11-diene	P	A	[162]

227	5β-hydroperoxy-eudesma-4(15),11-diene	P	A	[23]
228	6,7-Dehydroartemisinic acid	P	A	[169]
229	6α-hydroxy-arteannuin J	P	A	[23]
230	7α-Dihydroxyamorph-4-ene 3-acetate	P	A	[135]
231	7α-hydroxy-artemisinic acid	P	A	[23]
232	Abeo-amorphane sesquiterpene	P	A	[23]
233	Amorph-4-en-7-ol	P	A	[170]
234	Amorphane epoxide,	P	A	[135]
235	Annulide	P	A	[137,160]
236	Ar-curcumene	A	P	[139,144]
237	Aromadendrene epoxide	P	A	[6]
238	Arteannoides A to E	P	A	[26]
239	Arteannoides F to R	P	A	[26]
240	Arteannoides U to Z	P	A	[39]
242	Arteannuin A	P	A	[152]
243	Arteannuin B	P	A	[171]
244	Arteannuin B	P	A	[6]
245	Arteannuin D	P	A	[136]
246	Arteannuin E	P	A	[152]
247	Arteannuin F	P	A	[29]
248	Arteannuin O	P	A	[167]
249	Arteannuin P	P	A	[28]
250	Arteannuin Q	P	A	[29]
251	Arteannuin S	P	A	[23]
252	Arteannuin T	P	A	[23]
253	Arteannuin U	P	A	[23]
254	Arteannuin V	P	A	[23]
255	Arteannuin W	P	A	[23]
256	Arteannuin Y	P	A	[23]
257	Arteannuin Z	P	A	[23]
258	Arteannuins H, I, J, K, L, M and N	P	A	[168]
259	Artemanin A	P	A	[29]
260	Artemanin B	P	A	[38]
261	Artemin	A	P	[172]
262	Artemisal	A	P	[66]
263	Artemisin	P	A	[173]
264	artemisinic acid	P	A	[158,172]
265	Artemisinic acid methyl ester	P	A	[168]
266	Artemisinic acid, 6α-peroxy ester	P	A	[23]
267	Artemisinic aldehyde	P	A	[160]
268	Artemisinin	P	A	[158]
269	Artemisinin B	P	A	[6]
270	Artemisinin G	P	A	[174]
271	Artemisinol	P	A	[175]
272	Artemisitene	P	A	[153]
273	Artesin	A	P	[36]
274	Berbenome	A	P	[139]
275	Bicycloelemene	A	P	[139]
276	Bicycloelemene	A	P	[139]
277	Bicyclogermacrene	P	P	[139,145]
278	Calamenene	A	P	[139]
279	Caryophylladienol I	P	A	[137]

280	Caryophylladienol II	P	P	[137]
281	Caryophyllene oxide	P	P	[121,126]
282	Cedra-8-en-13-ol, acetate	P	A	[137]
283	Cedra-8(15)-en-9α-ol	P	A	[137]
284	Cedra-8(15)-en-9α-ol acetate	P	A	[137]
285	Cedrol	P	A	[147,152]
286	Cedryl acetate	P	A	[137]
287	Chamazulene	A	P	[149]
288	*cis*-Arteannuic alcohol	P	A	[137]
289	*cis*-Calamenene	P	A	[137]
290	*cis*-Caryophyllene oxide	P	A	[137]
291	*cis*-Lanceol	P	A	[167]
292	Cubenol	P	A	[176]
293	Cuminaldehyde	A	P	[139]
294	Cyclocolorenone	P	A	[164]
295	Cycloprop[7,8]azuleno[3a,4 -b]oxirene, decahydro- 1,4a,7,7-tetramethyl-, (1*R*,6a*R*,7a*R*,7b*S*)	P	A	[6]
296	d-myrthenal	A	P	[139]
297	Davanone	A	P	[149]
298	Dehydroarteannuin L	P	A	[23]
299	Dehydroartemisinin	P	A	[177]
300	Deoxyarteannuin B	P	A	[169]
301	Deoxyartemistene	P	A	[23]
302	Dihydroarteannuin B	P	A	[168]
303	Dihydroartemisinic	P	A	[168]
304	Dihydroartemisinic acid hydroperoxide	P	A	[65]
305	Dihydroartemisinic alcohol	P	A	[160]
306	Dihydroartemisinic aldehyde	P	A	[178]
307	Dihydroxy-amorphane	P	A	[168]
308	Dihydroxycadinanolide	P	A	[179]
309	Dihyro-epi-deoxyarteannuin B	P	A	[162]
310	Elemol	P	A	[137]
311	Elemyl acetate	P	A	[140]
312	Epi-11-hydroxy-arteannuin I	P	A	[23]
313	Epi-Cubenol	P	A	[159]
314	Epi-deoxyarteannuin B	P	A	[169]
315	Epi-globulol	P	P	[139,150]
316	Farnesal	P	A	[164]
317	Farnesol	P	A	[136,160]
318	Farnesyl pyrophosphate	P	A	[160]
319	Germacrene B	P	A	[122]
320	Germacrene D	p	p	[139,152]
321	Germacrene D-4-ol	P	P	[139,144]
322	Globulol	P	P	[139,140]
323	Humulene epoxide I	P	A	[170]
324	Humulene epoxide II	P	A	[137,147]
325	Intermedeol	A	P	[139]
326	Intermediol	A	P	[139]
327	Isoalantolactone	A	P	[180]
328	Isoannulide	P	A	[168]
329	Isoarteannuin A	P	A	[23]

330	Isopropyl-3- methylbenzene	A	P	[145]
331	Kongol	P	A	[139]
332	Ledol	P	A	[40]
334	Maritimin	A	P	[172]
335	Nor-amorphane	P	A	[162]
336	Norannuic acid	P	A	[152]
337	Norannuic acid (bis-nor-amorphane)	P	A	[152]
338	Norannuic acid formyl ester	P	A	[162]
339	Norsantolinifolide	A	P	[172]
340	Occidentalol	P	A	[137]
341	Occidentalol acetate	P	A	[137]
342	Occidol	P	A	[137]
343	Oxabicyclo[4.1.0]heptane, 4-(1,5-dimethyl-4-hexen-1- ylidene)-1-methyl-, (1*R*,4*Z*,6*S*)-	A	P	[141]
344	p-isopropyl phenol	A	P	[139]
345	Pregeijerene	P	A	[159]
346	Santolinifolide A	A	P	[172]
347	Selina-4,11-diene	P	A	[160]
348	*t*-Muurolol	P	P	[140]
349	Taurin	A	P	[172]
350	Trans- β-selinene	P	A	[162]
351	Trans-Arteannuic alcohol	P	A	[137]
352	Trans-caryophyllene	P	P	[135,139]
353	Trans-Nerolidol	P	A	[140]
354	Trans-α-bergamotol	A	P	[139]
355	Trans-α-bergamotol	A	P	[139]
356	Trans-β-farnesene	p	p	[139,155]
357	Tricosane	A	P	[154]
358	Umbellulone	A	P	[66]
359	Verboccidentene	P	A	[170]
360	Vetivazulene	A	P	[181]
361	α-Aromadendrene	P	A	[136]
362	α-Copaene	P	P	[139,144]
363	α-epoxy-arteannuic acid	P	A	[161]
364	α-Epoxyartemisinic acid	P	A	[168]
365	α-Farnesene	P	A	[182]
366	α-Gurjunene	P	A	[152]
367	α-Humulene	p	p	[139,146]
368	α-Hydroxysantonin	P	A	[183]
369	α-Selinene	p	P	[139,151]
370	β-Cadinene	P	A	[152]
371	β-Caryophyllene	p	p	[139,176]
372	β-Costol	A	P	[139]
373	β-Eudesmo	P	A	[170]
374	γ-Cadinene	P	A	[137]
375	γ-Cadinol	P	A	[183]
376	γ-Eudesmol	P	A	[137]
377	**γ**-Muurolene	P	A	[137]
378	γ-Selinene	P	A	[170]
379	δ-cadinene	P	A	[161]

P = Present, A = Absent

Table S3: Polyphenols from *A. annua* and *A. afra*

N	Compound	*A. annua*	*A. afra*	Reference
380	1-Caffeoyl-5-feruoylquinic acid	P	P	[184]
381	2-Methoxy-3-(2-propenyl) phenol	P	A	[136]
382	3-Allyl-6-methoxyphenol	P	A	[153]
383	3-Caffeoyl-4-feruloylquinic acid	P	P	[184]
384	3-Caffeoyl-5-feruloylquinic acid	P	P	[184]
385	3-Feruloyl-5-caffeoylquinic acid	P	P	[184]
386	3-p-O-coumaroyl-5-O-caffeoylquinic acid	P	A	[10]
387	3,3,5,6,7-Pentahydroxy-3-4-dimethoxyflavone	P	A	[185]
388	3,3′4′,7,7-Pentahydroxyflavone, 3-O-6-D-Glucopyranoside.	P	A	[185]
389	3,4-Diferuoylquinic acid	P	P	[184]
390	3,5-Di-O-caffeoylquinic acid	P	P	[24]
391	methyl-3,4-di-O-caffeoylquinic acid	P	A	[185]
392	3,6'-O-diferuloylsucrose	P	A	[185]
293	3,5-Dicaffeoylquinic acid	P	P	[29], [134]
394	5'-β-D-glucopyranosyloxyjasmonic acid	P	A	[185]
395	3,5-Diferuoylquinic acid	P	P	[184]
396	3,7-Dirhamnoside patuletin	P	A	[17]
397	3′,5,7,8-Tetrahydroxy-3,4′-dimethoxyflavone	P	A	[185]
398	4-Caffeoyl-5-feruloylquinic acid	P	P	[184]
399	4-Feruloyl-5-caffeoylquinic acid	P	A	[184]
400	4,5-Diferuoylquinic acid	P	P	[184]
401	4,5-Diferuoylquinic acid	P	P	[184]
402	4′-*O*-methylgenkwanin	A	P	[186]
403	*4H*-1-Benzopyran-4-one 5-hydroxy-2-(2-hydroxy-3,4- di-methoxyphenyl)-3,7-dimethoxy	P	A	[6]
404	5-hydroxy- 3,6,7,40-tetramethoxyflavone	P	A	[187]
405	5, 7, 4′- trimethoxy-8,3″-dihydroxyflavone	P	A	[40]
406	5,3′-Dihydroxy, 3,6,7,5′-tetramethoxyflavone	P	A	[5]
407	7-Methoxyacacetin	A	P	[185]
408	7-Methoxyacacetin	A	P	[184]
409	Acacetin	A	P	[184]
410	Iso-chlorogenic acid B	P	P	[184]
411	Anethole	P	A	[137]
412	Apigenin	P	P	[184,149]
413	Apigenin-6-C-hexoside-8-C-pentoside.	P	A	[10]
414	Artemetin	P	A	[6]
415	Artemetin			[188]
416	Artemisiannuside A	P	A	[105]
417	Astragalin	P	P	[163]
418	Axillarin	P	A	[189]
419	Axillarin	P	A	[186]
420	Benzyl cinnamate	P	A	[6]
421	Bonanzin	P	A	[33]
422	Caffeic acid	P	P	[48]
423	Caffeoylcoumaroyltartaric acid	P	A	[10]

424	Casticin	P	A	[190]
425	Casticin	P	A	[190]
426	Chlorogenic acid	P	P	[29,48]
427	Chrysoeriol	P	P	[184,149]
428	Chrysoeriol rutinoside	P	A	[31]
429	Chrysosplenetin	P	P	[190]
430	Chrysosplenol C	P	P	[6]
432	Chrysosplenol D	P	P	[6]
433	Chrysosplenol E	P	P	[6]
434	Cirsilineol	P	P	[190]
435	Cirsiliol	P	A	[190]
436	Cirsimaritin	P	A	[190]
437	Cirsimaritin	P	A	[190]
438	Coumaric acid	P	P	[6]
439	Cynaroside	P	A	[186]
440	Dihydroxy-dimethoxyl-O-hexoside	P	A	[10]
441	Diosmetin	A	P	[184]
442	Eleutheroside B	P	A	[10]
443	Eriodictyol-7-O-hexoside.	P	A	[10]
444	Esculetin	P	P	[10]
445	Eugenol	P	A	[140]
446	Eugenyl isovalerate	P	A	[6]
447	Eupalitin	P	P	[33]
448	Eupatin	P	P	[33]
449	Eupatorin	P	A	[190]
450	Fisetin	P	A	[10]
451	Genkwanin	A	P	[184]
452	Glucoluteolin	P	A	[190]
453	Iso-kaempferide	P	A	[186]
454	Isochlorogenic acid	P	P	[184]
455	Isochlorogenic acid A	P	P	[184]
456	Isochlorogenic acid C	P	P	[184]
457	Isoquercitrin	P	A	[190]
458	Isorhamnetin	P	P	[190]
359	Isorhamnetin	P	P	[190]
460	Isorhamnetin 3-glucoside	P	P	[190]
361	Isorhamnetin-O-hexoside	P	A	[10]
462	kaempferol	p	P	[186,187]
463	Kaempferol-6-methoxy glucoside	P	P	[186]
464	Luteolin	p	P	[192,187]
465	Luteolin 7-O-pentoside	P	A	[33]
466	Luteolin-7- methyl ethe	P	P	[190]
467	Mearnsetin	P	A	[184]
468	Mearnsetin- glucoside	P	A	[184]
469	Methyl cinnamate	P	A	[6]
370	Methyl eugenol	P	A	[137]
471	Mikanin	P	A	[184]
472	Ouercimeritrin	P	A	[186]
473	*p*-Allylanisole	P	A	[6]
474	Pachypodal	P	A	[167]
475	Patuletin	P	A	[190]

476	Patuletin-3-*O*-glucoside	P	A	[190]
477	Penduletin	P	P	[195]
478	Quercetagetin 3,4′-dimethyl -ether	P	A	[190]
579	Quercetagetin-3-methylether	P	A	[190]
480	Quercetagetin-3,4′- dimethyl ether	P	A	[189]
481	Quercetagetin-4-methylether	P	A	[190]
482	Quercetagetin-6,7,3′,4′- tetramethylether	P	A	[6]
483	Quercetin	p	P	[187]
484	Quercetin	P	P	[190]
485	Quercetin 3-rutinoside	P	P	[190]
486	Quercetin-3-glucoside	P	P	[190]
487	Quercetin-3-methylether	P	P	[6]
488	Quercetin-3′-glucoside	P	P	[190]
489	Quercimeritrin	P	A	[190]
490	Quinic acid	P	P	[184]
491	Retusin	P	P	[168]
492	Rhamnetin	P	P	[190]
493	Rhamnocitrin	P	A	[190]
494	Rosmarinic acid	P	A	[34]
494	Rutin	P	P	[24,186]
495	Tamarixetin	P	P	[184,190]
496	Tamarixetin	P	A	[190]
497	Vitexin (8-C-glucosyl apigenin)	P	P	[191]

P = Present, A = Absent

Table S4: Triterpenes from *A. annua* and *A. afra.*

N	Compound	*A. annua*	*A. afra*	Reference
498	Baurenol	P	A	[153]
499	Betulinic acid	P	A	[167]
500	Daucosterol	P	A	[6]
501	Friedelan-3-β-ol	P	A	[171]
502	Friedelin	P	P	[172,191]
503	Oleanolic acid	P	A	[153]
504	Squalene	A	P	[170]
505	Stigmasterol	P	A	[6]
506	Taraxasterone	P	A	[153]
507	Taraxerol acetate	P	A	[17]
508	α-Amyrenone	P	A	[153]
509	α-Amyrin	P	P	[153,191]
510	β-Amyrin	P	P	[153,191]
511	β-Amyrin acetate	P	A	[171]
512	β -Fridelan-3-ol	P	A	[6]
513	β-Sitosterol	P	A	[6]

P = Present, A = Absent

Table S5: Coumarins from *A. annua* and *A. afra.*

N	Compound	*A. annua*	*A. afra*	Reference
514	(±)-Qinghaocoumarin A	P	A	[33]

515	12-Hydroxy-α-cyperone	P	P	[172,192]
516	2,2-Dihydroxy-6-methoxy-*2H*-1- benzopyran	P	A	[6]
517	2,2,6-Trihydroxychromene	P	A	[6]
518	2,4-Di- hydroxy-6-methoxyacetophenone	P	A	[151]
519	5-Nonadecylresorcinol 3-O-methyl ether	P	A	[6]
520	6,7-Dimethoxydihydrocoumarin	P	A	[6]
521	Cis-melilotoside	P	A	[32]
522	Trans-melilotoside	P	A	[32]
523	Coumarin	P	P	[6]
524	Eleutheroside B	P	A	[193]
525	Esculetin	P	P	[194]
526	Iso-Fraxidin	P	P	[172,180,187]
527	Ouebrachitol	A	P	[195]
528	Qinghaocoumarin B	P	A	[192]
529	Sacoparone	P	A	[6]
530	Scopoletin	P	P	[187,193]
531	Scopolin	P	P	[24]
532	Tomentin	P	A	[29]
533	Trimethoxy-coumarin			[31]
534	Umbelliferone derivatives	A	P	[6]

P = Present, A = Absent

Table S6: Diterpenes from *A. annua* and *A. afra*

N	Compound	*A. annua*	*A. afra*	Reference
535	(2*E*)-Hexadecene	P	A	[19]
536	13-*epi*-manool (8(17),14- labdadien-13-ol	P	A	[137]
537	7R,11R-phytol	P	A	[135]
537	8(14),15-isopimaradiene	P	A	[137]
538	Abscisic acid	P	A	[194]
539	Isophytol	P	A	[137]
540	Phytene-1-ol-2-hydroperoxide	P	A	[135]
541	Phytene-1,2-diol	P	A	[6]
542	Phytol	P	P	[135,167]
543	Phytone	P	A	[136]

P = Present, A = Absent

Table S7: Guaianolides from *A. annua* and *A. afra.*

N	Compound	*A. annua*	*A. afra*	Reference
544	11,13-Dihydromatricarin	A	P	[17]
545	Guaianolides 1 (2 derivatives)	A	P	[170]

546	Guaianolides 2 (6 derivatives)	A	P	[173,17]
547	Guaianolides 3 (11 derivatives)	A	P	[161,174]
548	Guaianolides 4 (2 derivatives)	A	P	[170]
549	Guaianolides 5 (3 derivatives)	A	P	[170]
550	1α,4α-Dihydroxybishopsolicepolide	A	P	[14]
551	Yomogiartemin	A	P	[14]

P = Present, A = Absent

Table S8. Simple Aryl Ketones from *A. annua* and *A. afra.*

N	Compound	*A. annua*	*A. afra*	Reference
552	2-Hydroxy-4,6-dimethoxyacetophenone	P	A	[6]
553	2,4-dihydroxy-6-methoxyacetophenone	A	P	[36]
554	2′,4′-dihydroxy-6′- methoxyacetophenone	P	A	[6]
555	Annphenone	P	A	[151]
556	Domesticoside	P	A	[195]
557	*p*-Hydroxyacetophenone	A	P	[36]

P = Present, A = Absent

Table S9: Glaucolides from *A. annua* and *A. afra.*

N	Compound	*A. annua*	*A. afra*	Reference
558	12-Hydroxy-α-cyperone	A	P	[170]
559	1α-Hydroxyafraglaucolide	A	P	[170]
560	1α-Hydroxyisoafraglaucolide	A	P	[170]
561	1β-Hydroxyafraglaucolide	A	P	[170]
562	Artemisia glaucolide	A	P	[170]
563	Eudesmaafraglaucolide glaucolide 7	A	P	[173,17]

P = Present, A = Absent

Table S10: Long chain alkanes from *A. annua* and *A. afra.*

N	Compound	*A. annua*	A. *afra*	Reference
564	Cerylcerotinate	A	P	[191]
565	Dodecane	P	A	[6]
566	Heneicosane	P	A	[137]
567	Hexacosane	P	A	[137]
568	Hexadecane	P	A	[6]
569	Nonacosane	A	P	[191]
570	Nonadecane	P	A	[137]
571	Pentacosane	P	A	[137]
572	Tetracosane	P	A	[137]
573	Tetratriacontane	P	A	[146]
574	Triacontane 6-triacontanone wax ester	A	P	[188]
575	Tridecane	P	A	[6]
576	Wax ester	A	P	[191]

P = Present, A = Absent

Table S11: Other compounds from *A. annua* and *A. afra.*

N	Compound	*A. annua*	*A. afra*	Reference
577	(R)-15,16-Didehydrocoriolic acid	P	A	[105]

578	Homoeriodictyol	P	A	[105]
579	Qinghaosu I and III	P	A	[35]
580	Cadinanolide	P	A	[196]
581	n-heptadecanyl-ß-D-glucopyronoside	P	A	[150]
582	n-Cos-(Z)-9-enoic acid.	P	A	[150]
583	n-Cos-(Z)-10-enoic acid	P	A	[150]
584	n-Nonacosanyl n-octadec-9, 12-dienoate.	P	A	[150]
585	n-Heptadecanyl linoleate	P	A	[150]
586	1-Octacosanol	P	A	[150]
587	Isodocosanol	P	A	[150]
588	Isononadecano	P	A	[150]
589	Qinghaolignan A.	P	A	[86]
590	Qinghaolignan B	P	A	[86]
591	3,5-Cycloheptadienone	P	A	[6]
592	5-Methyl-2-furancarboxyaldehyde	P	A	[86]
593	Hexylcyclohexane	P	A	[137]
594	Jasmone	P	A	[141]
595	2,5-Dihydro-3-methylfuran	P	A	[138]
596	Anisole	P	A	[6]
597	Benzyl valerate	P	A	[136]
598	Isoamyl salicylate	P	A	[6]
599	Phenylpropanoic acid	P	A	[135]
600	2-Methylbutanoic acid	P	A	[6]
602	3-Methylbutana	P	A	[6]
603	(Z)-2-Nonenal	P	A	[138]
604	Benzyl isovalerate	P	A	[136]
605	Amyl 2-methylbutyrate	P	A	[137]
606	2-Benzyloctanal	P	A	[6]
607	3-Hexenyl butanoate	P	A	[136]
608	Trans-2,4-Hexadiene	P	A	[125]
**609**	vitexnegheteroin M	P	A	[197]
**610**	sibricose A5	P	A	[197]
**611**	securoside A	P	A	[197]
**612**	citrusin D	P	A	[197]

P = Present, A = Absent
